# Identification of potential genes related to breast cancer brain metastasis in breast cancer patients

**DOI:** 10.1042/BSR20211615

**Published:** 2021-10-12

**Authors:** Lijian Zhang, Luxuan Wang, Hua Yang, Chunhui Li, Chuan Fang

**Affiliations:** 1 Postdoctoral Research Station of Neurosurgery, Affiliated Hospital of Hebei University, Hebei University, Baoding, Hebei, China; 2 Department of Neurosurgery, Affiliated Hospital of Hebei University, Hebei University, Baoding, Hebei, China; 3 Key Laboratory of Precise Diagnosis and Treatment of Glioma in Hebei Province, Affiliated Hospital of Hebei University, Hebei University, Baoding, Hebei, China; 4 Department of Neurology, Affiliated Hospital of Hebei University, Hebei University, Baoding, Hebei, China; 5 Department of Oncology, Affiliated Hospital of Hebei University, Hebei University, Baoding, Hebei, China

**Keywords:** bioinformatics, Brain metastasis, tumor microenvironments

## Abstract

Brain metastases (BMs) usually develop in breast cancer (BC) patients. Thus, the molecular mechanisms of breast cancer brain metastasis (BCBM) are of great importance in designing therapeutic strategies to treat or prevent BCBM. The present study attempted to identify novel diagnostic and prognostic biomarkers of BCBM. Two datasets (GSE125989 and GSE100534) were obtained from the Gene Expression Omnibus (GEO) database to find differentially expressed genes (DEGs) in cases of BC with and without brain metastasis (BM). A total of 146 overlapping DEGs, including 103 up-regulated and 43 down-regulated genes, were identified. Functional enrichment analysis showed that these DEGs were mainly enriched for functions including extracellular matrix (ECM) organization and collagen catabolic fibril organization. Using protein–protein interaction (PPI) and principal component analysis (PCA) analysis, we identified ten key genes, including *LAMA4, COL1A1, COL5A2, COL3A1, COL4A1, COL5A1, COL5A3, COL6A3, COL6A2*, and *COL6A1*. Additionally, *COL5A1, COL4A1, COL1A1, COL6A1, COL6A2*, and *COL6A3* were significantly associated with the overall survival of BC patients. Furthermore, *COL6A3, COL5A1*, and *COL4A1* were potentially correlated with BCBM in human epidermal growth factor 2 (HER2) expression. Additionally, the miR-29 family might participate in the process of metastasis by modulating the cancer microenvironment. Based on datasets in the GEO database, several DEGs have been identified as playing potentially important roles in BCBM in BC patients.

## Background

Breast cancer (BC) is the most commonly diagnosed cancer in women [1]. The central nervous system (CNS) is one of the most common sites to which BC usually spreads, along with the bones, lungs, and liver. Metastasis of cancer to the brain has potentially devastating clinical consequences, resulting in an estimated survival time of less than 1 year, despite recent breakthroughs in neurologic therapies [2]. CNS metastases occur as a distant recurrence of BC. When cancer cells metastasize to the brain, patients’ prognoses are poor, and they have few therapeutic options [3]. Surgical resection of single brain metastasis (BM) is considered a standard treatment for patients with metastases in accessible locations, good functional status, and absent/controlled extracranial disease. Overall surgical mortality is approximately 0.7–1.9%, while neurological morbidity is 3.9–6% [4]. The rate of postoperative complications, including pneumonia, urinary infections, and venous thrombosis, is approximately 13.9% [5,6]. Further, chemotherapy drugs targeting BC cells in the brain are not effective, because they cannot cross the blood–brain barrier [7,8]. In light of the poor outcomes and limited treatment options following a diagnosis of breast cancer brain metastasis (BCBM), a better understanding of the mechanisms may help improve clinical decision-making. The development of novel biomarkers for BCBM would not only provide deeper insights into its pathology but would also help provide sensitive and efficient approaches for diagnosis and treatment.

Previous studies have documented that tumor BM is a complicated process, including steps such as the detachment of cells from the primary tumor, invasion of the extracellular matrix (ECM), travel through the bloodstream to arrest at a secondary site, and extravasation from the vasculature to establish metastasis in a new organ [9]. Numerous animal experiments and clinical trials appear to support Paget’s ‘seed and soil’ hypothesis for non-random metastatic spread [10,11]. Although the initial steps for the metastasis of primary tumor cells may significantly overlap, the circulation patterns, extravasation barriers, and potential to survive in foreign tissue varies with the molecular subtype of the primary tumor [12]. Molecular cross-talk between primary BC and other cells in the surrounding microenvironment, as well as the role of primary BC cells in modulating the brain microenvironment during metastasis, are also key factors driving metastatic outgrowth [13,14]. Evidence has shown that, prior to reaching the brain, some metastatic primary BC cells already express proteins that are essential for the establishment of brain metastases, including serpins [15], matrix metalloproteases [16], and αB-crystallin [17]. BCBM cells overexpress bone morphogenic proteins (BMPs) to promote the differentiation of neural progenitor cells (NPCs) into astrocytes and create a permissive microenvironment which allows cancer cells to colonize and proliferate [18]. Until now, extensive work in identifying driver genes of BCBM has provided some potential explanations for its metastasis to the brain. However, the underlying mechanisms involved in the invasion and metastatic outgrowth of BC cells into the CNS are largely unknown, and a novel option for the prevention or treatment of BM is unavailable [19].

With the development of genomics and bioinformatics, large amount of BC-related data are publicly available in several databases. Bioinformatics is a useful tool for extracting valuable information from existing data at a relatively low cost, and can help identify novel diagnostic biomarkers of BCBM [20,21]. In this study, by screening differentially expressed genes (DEGs) between BC patients with and without BM in two individual Gene Expression Omnibus (GEO) datasets, we aimed to explore potentially important genes associated with BCBM, and to investigate more information about the microenvironmental influence on BCBM development. The present study may help contribute to preventing BCBM at early stages in the future.

## Materials and methods

### Microarray data

Datasets were obtained from the National Center of Biotechnology Information (NCBI) GEO (https://www.ncbi.nlm.nih.gov/geo/) database. Two datasets (GSE125989 [22] and GSE100534 [23]) were used in the present study. The samples in the GSE125989 and GSE100534 datasets were detected using the Affymetrix Human Genome U133A 2.0 Array and the Affymetrix Human Gene 1.0 ST Array, respectively.

### Data processing

The GEO2R (http://www.ncbi.nlm.nih.gov/geo/geo2r/) based on R language limma package was used to analyze the gene expression data and to identify DEGs [24]. Those genes with |logFC| > 1 and *P*-values <0.05 were selected as DEGs (BC without BM vs. BCBM). There are 16 BC samples and 16 BCBM samples in the GSE125989 dataset, and there are 16 BC samples and 3 BCBM samples in the GSE100534 dataset (after removing the primary meningioma samples). To visualize the overlapping DEGs in both GSE125989 and GSE100534, a Venn diagram was generated using a freely accessible online tool (http://bioinformatics.psb.ugent.be/webtools/Venn/).

### Bioinformatics analysis of DEGs

In the Gene Ontology (GO) database, gene functions are divided into three parts, including cellular components (CCs), biological processes (BPs), and molecular functions (MFs). The Kyoto Encyclopedia of Genes and Genomes (KEGG) is a database integrating genome, chemistry, and system function information, which aims to reveal the genetic and chemical blueprint of life. It is a system function knowledge base that is based on computable experiments. We used the The Database for Annotation, Visualization and Integrated Discovery (DAVID) database for GO and KEGG enrichment analyses (http://david.abcc.ncifcrf.gov/). Functional protein–protein interaction (PPI) analysis is important for interpreting the potential molecular mechanisms of key cellular activities in pathogenicity. The Search Tool for the Retrieval of Interacting Genes (STRING) database (http://string-db.org) was used to construct PPI networks of high and low expression genes. An interaction score of 0.4 was treated as the cut-off criteria, and Cytoscape (http://www.cytoscape.org/) was used to visualize the PPI network. Hub genes with connection degrees > 9 were selected. Then, the Molecular Complex Detection (MCODE) in the Cytoscape software was used to obtain the modules within the PPI network with MCODE scores > 2 and number of nodes > 4. Functional enrichment analysis of the genes in each module was also completed using Metascape [25]. Finally, a principal component analysis (PCA) model was visualized for clustering trends based on the variation in the phase of injury using the ‘principal’ function in the R software [26].

### Transcription factors and expression correlation analyses

To elucidate the selected DEGs expression regulation on a systematic level, a transcription factor (TF)–miRNA coregulatory network was generated using NetworkAnalyst based on the TF-miRNA coregulatory interaction database. The expression correlation analysis was conducted using Gene Expression Profiling Interactive Analysis (GEPIA), which was based on available The Cancer Genome Atlas (TCGA) datasets. Pearson correlation coefficients and *P*-values were calculated (http://gepia.cancer‐pku.cn/index.html) [27].

### Survival analysis

To evaluate the effects of hub genes on the overall survival of BC patients, the publicly available Kaplan–Meier Plotter (http://kmplot.com/analysis/) was used. According to the median expression levels, the patients were classified into high and low expression groups [28]. Differences between the high and low expression groups were assessed using log‐rank tests. *P*<0.05 was considered to indicate statistically significant difference.

### Construction of BCBM prediction model

Here, we classified all samples into two sets, namely the samples with BM and samples without BM, adopted the expression of genes as a continuous predictor variable by glm package in R software [29]. We set the expression level of the genes as an independent variable, and whether it developed BM as the dependent variable to construct the logistic regression model. Firstly, the expression levels of COL4A1, COL5A1, and COL6A3 were extracted from GSE125989 and GSE100534 datasets. In each dataset, z-score normalization (∼N (0,1) normal distribution) was conducted, and then we merged the two datasets. Subsequently, the z-scores of these genes were used for building a logistic regression prediction model to predict whether BM would develop in BC patients. And the accuracy of the model was determined by receiver operating characteristic (ROC) curve.

## Results

### Identification of DEGs related to BCBM in BC patients

**Figure 1:**
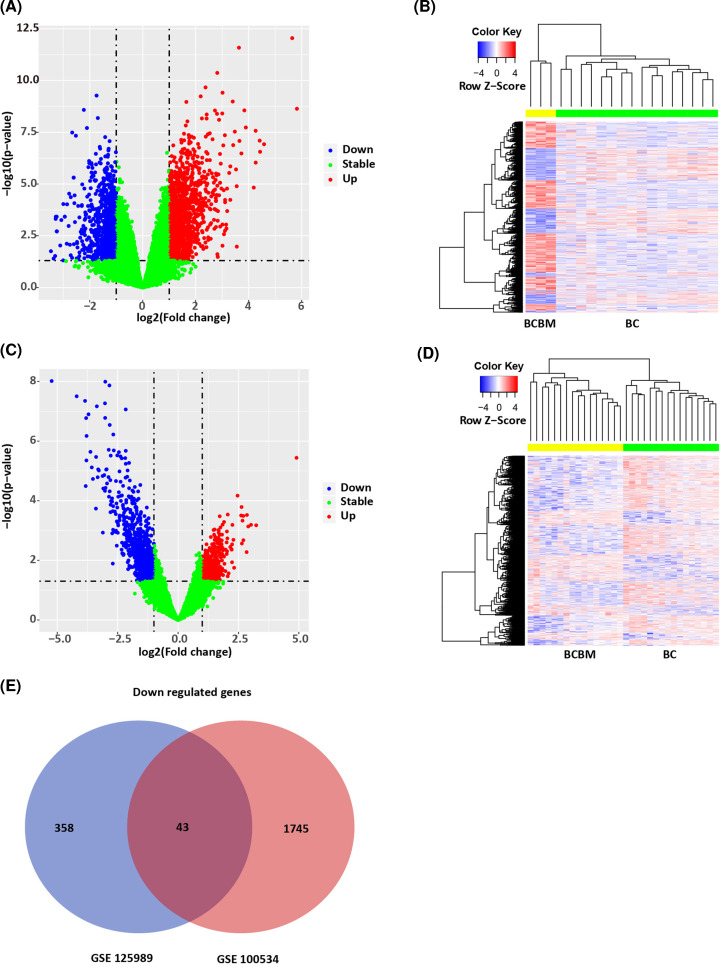
Identification of DEGs related to BCBM in BC patients (**A**) Volcano map of DEGs in GSE100534. X-axis: log_2_FC; Y-axis: −log_10_ (FDR). Blue: down-regulated genes; red: up-regulated genes; green: non-significant. (**B**) Heat map of DEGs in GSE100534. X-axis: sample; Y-axis: different genes. Red: high expression; blue: low expression. BCBM and BC samples could be obviously clustered into two clusters. (**C**) Volcano map of DEGs in GSE125989. X-axis: Log_2_FC; Y-axis: −log_10_ (FDR). Blue: down-regulated genes; red: up-regulated genes; green: non-significant. (**D**) Heat map of DEGs in GSE125989. X-axis: sample; Y-axis: different genes. Red: high expression; blue: low expression. BCBM and BC samples could be obviously clustered into two clusters. (**E**) Venn diagram of overlapped DEGs between GSE100534 and GSE125989.

In order to screen the genes potentially associated with BCBM in BC patients, DEG analysis was performed between BC specimens (without BM) and BCBM specimens, which were conducted in GSE100534 and GSE125989, respectively. In the GSE100534 dataset, BCBM specimens were compared with BC specimens. A total of 2485 DEGs were obtained, including 971 up-regulated and 1593 down-regulated (Figure 1A,B). In the GSE125989 dataset, BCBM specimens were compared with BC specimens. A total of 1417 DEGs were obtained, including 984 up-regulated and 433 down-regulated (Figure 1C,D). A total of 146 overlapping genes (103 up-regulated and 43 down-regulated) were obtained between the two datasets (Figure 1E), which we further analyzed in our research.

### PPI network construction and Hub gene selection

**Figure 2:**
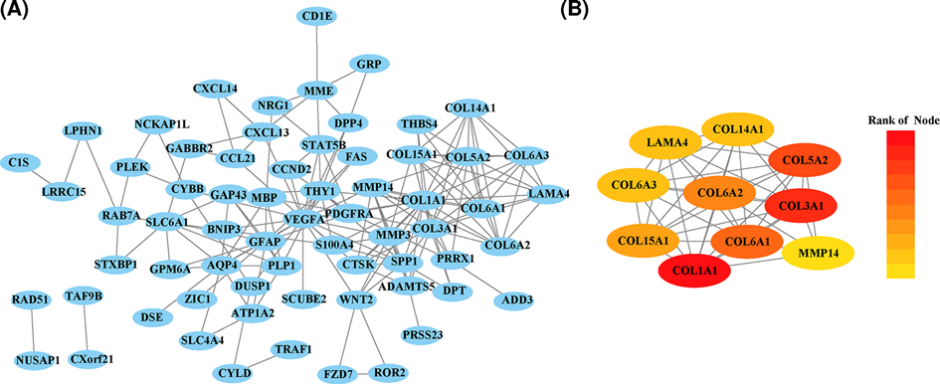
PPI network and top module of 146 DEGs (**A**) PPI network of DEGs in light blue and top one module in orange. (**B**) Top ten hub genes.

We next constructed a PPI network based on the 146 overlapping DEGs using the STRING database, and visualized it using Cytoscape software. There were 64 nodes and 174 edges in the PPI network (Figure 2A). Among these, the top ten genes with the highest interaction degrees were then identified, including Collagen Type I α 1 Chains (COL1A1), Collagen Type III α 1 Chains (COL3A1), Collagen Type V α 2 Chains (COL5A2), Collagen Type V α 3 Chains (COL5A3), Collagen Type VI α 1 Chains (COL6A1), Collagen Type VI α 2 Chains (COL6A2), Collagen Type VI α 3 Chains (COL6A3), Collagen Type XIV α 1 Chains (COL14A1), Collagen Type XV, α 1 (COL15A1), and Laminin Subunit α 4 (LAMA4) (Figure 2B).

### PCA of the 146 DEGs

**Figure 3:**
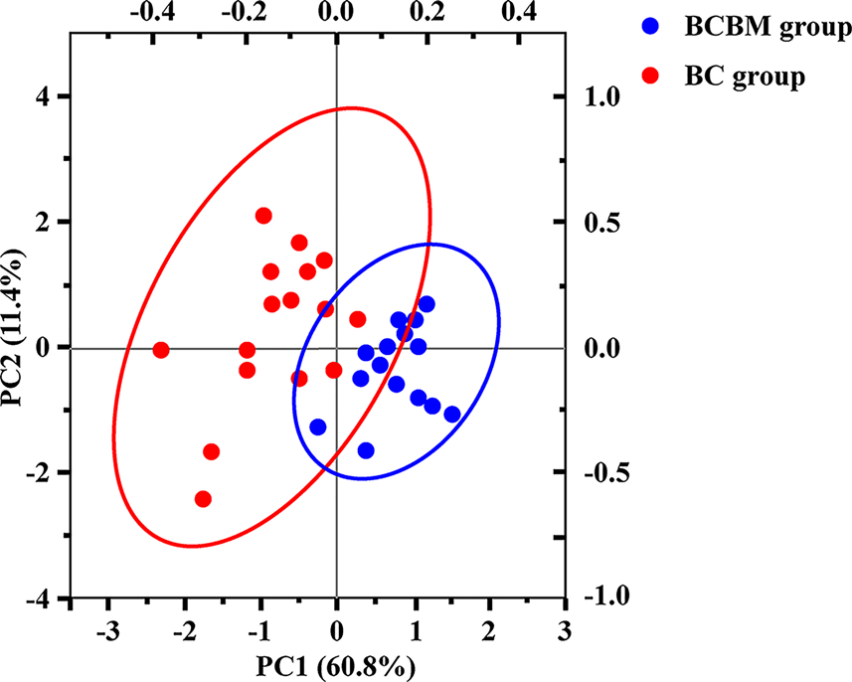
PCA of DEGs The variance in gene expression between BCBM group and BC group.

We performed PCA based on the expression profiles of DEGs to find DEGs that were highly related to the development of BCBM (Figure 3). Results showed that the first principal component (PC1) accounted for 60.8% of the total variance. The second principal component (PC2) accounted for an additional 11.4% of the total variance. Finally, changes in COL6A1, COL5A3, COL5A1, COL5A2, COL6A1, COL3A, COL6A1, COL14A1, and LAMA4 contributed the most to the separation between the BCBM and BC samples, which were also within top modules of the 146 DEGs. All of these genes are associated with ECM organization, the collagen catabolic process, and cellular adhesion.

### KEGG pathway and GO term enrichment analyses

**Figure 4:**
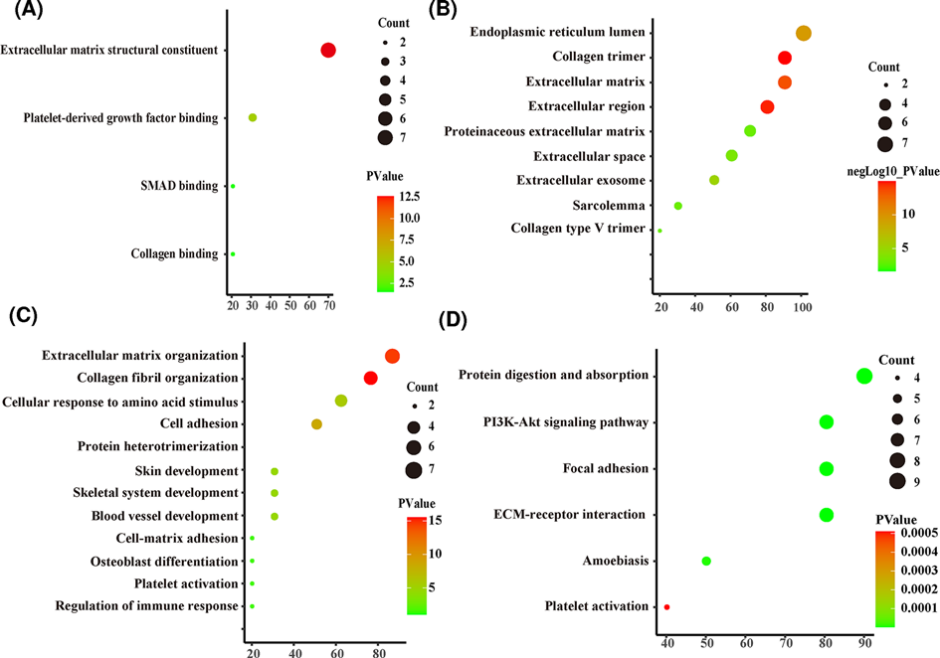
GO enrichment and KEGG pathway analysis of DEGs in BC and BCBM group (**A**) GO categories of MF. (**B**) GO categories of CC. (**C**) GO categories of BP. (**D**) KEGG pathway analysis of the DEGs.

We next performed functional enrichment analyses on the DEGs to obtain more information about the genes. Detailed results of GO enrichment analysis are shown in Table 1. The most significantly enriched MF terms were ECM organization, collagen catabolic process, and cellular adhesion (Figure 4A). The most noteworthy enriched CC terms were extracellular region and endoplasmic reticulum lumen ECM (Figure 4B). The most significantly enriched BP terms were ECM structural constituent, platelet-derived growth factor binding, and SMAD binding (Figure 4C). Detailed results of KEGG enrichment analysis are also shown in Table 2. The top six KEGG pathways are displayed in Figure 4D, including protein digestion and absorption, the PI3K-Akt signaling pathway, the Focal adhesion pathway, and ECM–receptor interactions.

**Table 1:** GO functional enrichment analysis for the DEGs

Term	Category	*P*-value	UniProt ID
**BP**			
Extracellular matrix organization	GO:0030198	2.66E-15	COL1A1, COL3A1, COL14A1, LAMA4, COL6A2, COL5A3, COL5A2, COL6A1, COL6A3
Collagen fibril organization	GO:0030199	3.10E-09	COL1A1, COL3A1, COL14A1, COL5A3, COL5A2
Cellular response to amino acid stimulus	GO:0071230	1.71E-06	COL1A1, COL3A1, COL5A2, COL6A1
Cell adhesion	GO:0007155	1.72E-06	COL1A1, COL15A1, LAMA4, COL6A2, COL6A1, COL6A3
Protein heterotrimerization	GO:0070208	2.32E-05	COL1A1, COL6A2, COL6A1
Skin development	GO:0043588	1.42E-04	COL3A1, COL5A3, COL5A2
Skeletal system development	GO:0001501	0.00229126	COL1A1, COL3A1, COL5A2
Blood vessel development	GO:0001568	0.020188218	COL1A1, LAMA4
Cell–matrix adhesion	GO:0007160	0.047226948	COL3A1, COL5A3
Osteoblast differentiation	GO:0001649	0.054392332	COL1A1, COL6A1
Platelet activation	GO:0030168	0.059988631	COL1A1, COL3A1
Regulation of immune response	GO:0050776	0.091476749	COL1A1, COL3A1
**MF**			
Extracellular matrix structural constituent	GO:0005201	2.59E-13	COL1A1, COL3A1, COL15A1, COL14A1, LAMA4, COL5A3, COL5A2
Platelet-derived growth factor binding	GO:0048407	1.39E-05	COL1A1, COL3A1, COL6A1
SMAD binding	GO:0046332	0.022698305	COL3A1, COL5A2
Collagen binding	GO:0005518	0.03154496	COL14A1, COL5A3
**CC**			
Endoplasmic reticulum lumen	GO:0005788	2.66E-15	COL1A1, COL3A1, COL15A1, COL6A2, COL5A3, COL5A2, COL6A1, COL6A3
Collagen trimer	GO:0005581	3.10E-09	COL1A1, COL3A1, COL14A1, LAMA4, COL6A2, COL5A3, COL5A2, COL6A1, COL6A3
Extracellular matrix	GO:0031012	1.71E-06	COL1A1, COL3A1, COL14A1, COL5A3, COL5A2
Extracellular region	GO:0005576	1.72E-06	COL1A1, COL3A1, COL5A2, COL6A1
Proteinaceous extracellular matrix	GO:0005578	2.32E-05	COL1A1, COL15A1, LAMA4, COL6A2, COL6A1, COL6A3
Extracellular space	GO:0005615	1.42E-04	COL1A1, COL6A2, COL6A1
Extracellular exosome	GO:0070062	0.00229126	COL3A1, COL5A3, COL5A2
Sarcolemma	GO:0042383	0.020188218	COL1A1, COL3A1, COL5A2
Collagen type V trimer	GO:0005588	0.047226948	COL1A1, LAMA4
Collagen type VI trimer	GO:0005589	2.98E-16	COL3A1, COL5A3

Category refers to the GO functional categories.

**Table 2:** Pathway enrichment analysis for the DEGs

Term	Category	*P*-value	UniProt ID
Protein digestion and absorption	hsa04974	4.62E-15	COL1A1, COL3A1, COL15A1, COL14A1, COL6A2, COL5A3, COL5A2, COL6A1, COL6A3
ECM–receptor interaction	hsa04512	1.43E-12	COL1A1, COL3A1, LAMA4, COL6A2, COL5A3, COL5A2, COL6A1, COL6A3
Focal adhesion	hsa04510	6.68E-10	COL1A1, COL3A1, LAMA4, COL6A2, COL5A3, COL5A2, COL6A1, COL6A3
PI3K-Akt signaling pathway	hsa04151	2.48E-08	COL1A1, COL3A1, LAMA4, COL6A2, COL5A3, COL5A2, COL6A1, COL6A3
Amoebiasis	hsa05146	6.33E-06	COL1A1, COL3A1, LAMA4, COL5A3, COL5A2
Platelet activation	hsa04611	5.10E-04	COL1A1, COL3A1, COL5A3, COL5A2

Category refers to the pathway functional categories.

### Survival analysis

**Figure 5:**
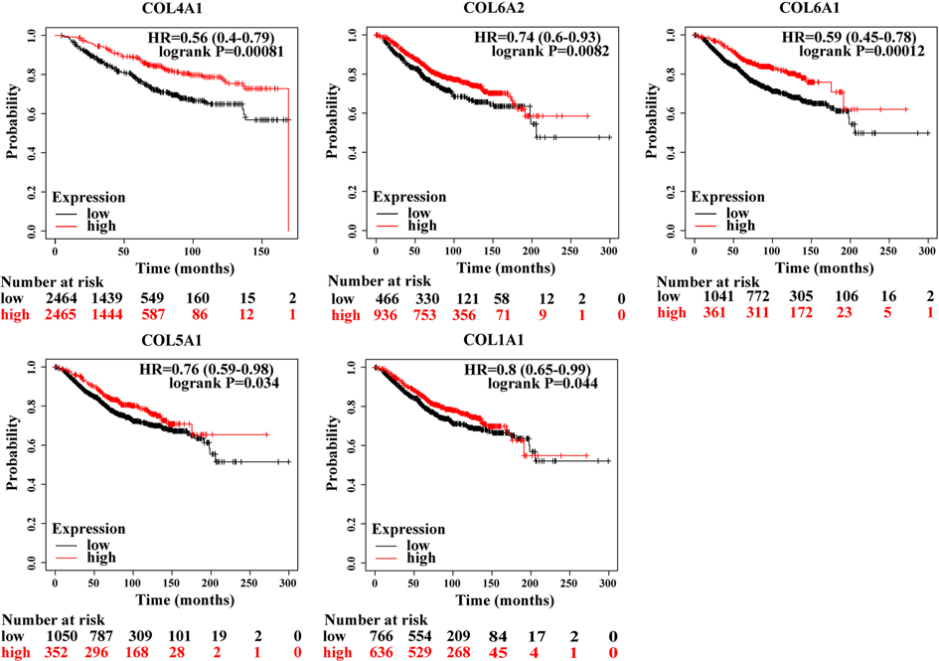
Prognostic value of ten genes

**Figure 6:**
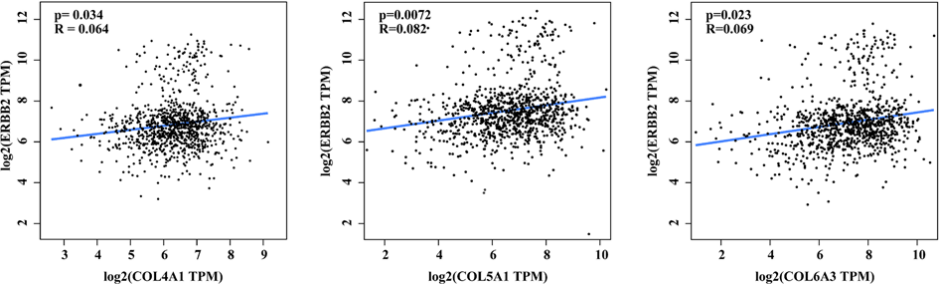
Correlation between HER2 and genes

To investigate the impacts of those ten hub genes on the survival rates of BC patients, we used Kaplan–Meier curve analysis. The median expression level was regarded as the cut‐off value for dividing the patients into two groups (high and low expression groups). Results showed that high expression of COL14A1 (HR = 0.56 [0.4–0.79], *P*=0.00081), COL6A2 (HR = 0.74 [0.6–0.93], *P*=0.0082), COL6A1 (HR = 0.59 [0.45–0.78], *P*=0.00012), COL5A1 (HR = 0.76 [0.59–0.98], *P*=0.034), and collagen type I α 1 chain (COL1A1) (HR = 0.8 [0.65–0.99], *P*=0.044) were associated with poor overall survival rates for BC patients. LAMA4, COL3A1, and COL5A1 expression had no significant influence on the overall survival rates of BC patients (*P*>0.05) (Figure 5). Moreover, correlation analysis results indicated that the expression of COL4A1, COL5A1, and COL6A3 was positively correlated with HER2 positive expression (Figure 6).

### TF regulatory network analysis of ten genes

**Figure 7:**
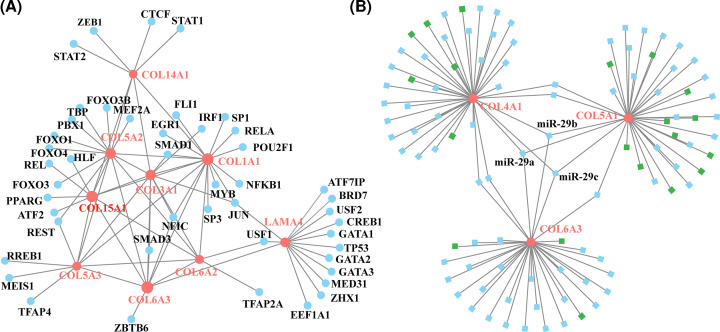
The TF regulatory network (**A**) The TF regulatory network of the top ten hub genes. Pink circle means the hub gene and blue circle means the transcription. (**B**) The TF regulatory network of selected DEGs (*COL4A1, COL5A1*, and *COL6A3*). The pink circle means the selected DEGs and blue square means the miRNAs.

We constructed a gene–TF regulatory network based on the ten identified genes—which included 56 interaction pairs between 10 genes and 45 TFs (Figure 7A). LAMA4 was found to be regulated by 11 TFs, COL1A1 was regulated by 10 TFs, COL5A2 was regulated by 8 TFs, COL3A1 was regulated by 5 TFs, COL4A1 was regulated by 4 TFs, COL5A1 was regulated by 4 TFs, COL5A3 was regulated by 4 TFs, COL6A3 was regulated by 3 TFs, and COL6A2 was regulated by 2 TFs. We further investigated the miRNAs that could regulate COL4A1, COL5A1, and COL6A3 (Table 3). Our findings suggested that the expression of these three genes was significantly correlated with each other, and that they were all also under the regulation of miRNA-29 family, including miR-29a, miR-29b, and miR-29c (Figure 7B).

**Table 3:** The potential TFs of ten hub genes

Genes	TFs	Counts
*LAMA4*	ATF71IP, BRD7, USF2, CREB1, GATA1, TP53, GATA2, GATA3, MED31, ZHX1, EEF1A1	11
*COL1A1*	SP3, JUN, MYB, NFKB1, POU2F1, RELA, SP1FLI1, EGR1, SMAD1, WFIC	10
*COL5A2*	MEF2A, FOXO3B, TBP, PBX1, FOXO1, FOXO4, FOXO3, REL	8
*COL3A1*	SMAD1, IRF1, NFIC, SMAD3, JUN	5
*COL4A1*	STAT2, ZEB1, CTCF, STAT1	4
*COL5A1*	IILF, REL, PPARG, ATF2	4
*COL5A3*	REST, RREB1, MEIS1, TFAP4	4
*COL6A3*	ZBTB6, SMAD3, WFIC	3
*COL6A2*	NFIC, TFAP2A	2
*COL6A1*	SMAD3	1

### Construction and validation of BCBM prediction model

**Figure 8:**
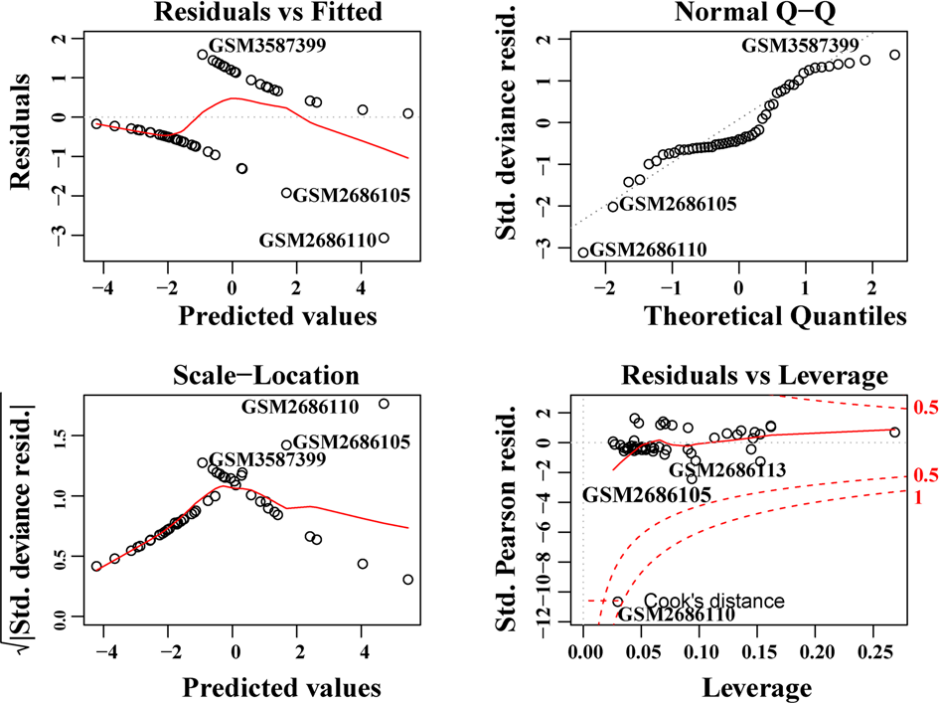
Model diagnosis diagram, Residuals vs. Leverage diagram The red dotted line presented the COOK distance, and the points with COOK distance > 0.5 were considered as influential points, which would affect the reliability of the model. There were no influential points in the model.

**Figure 9:**
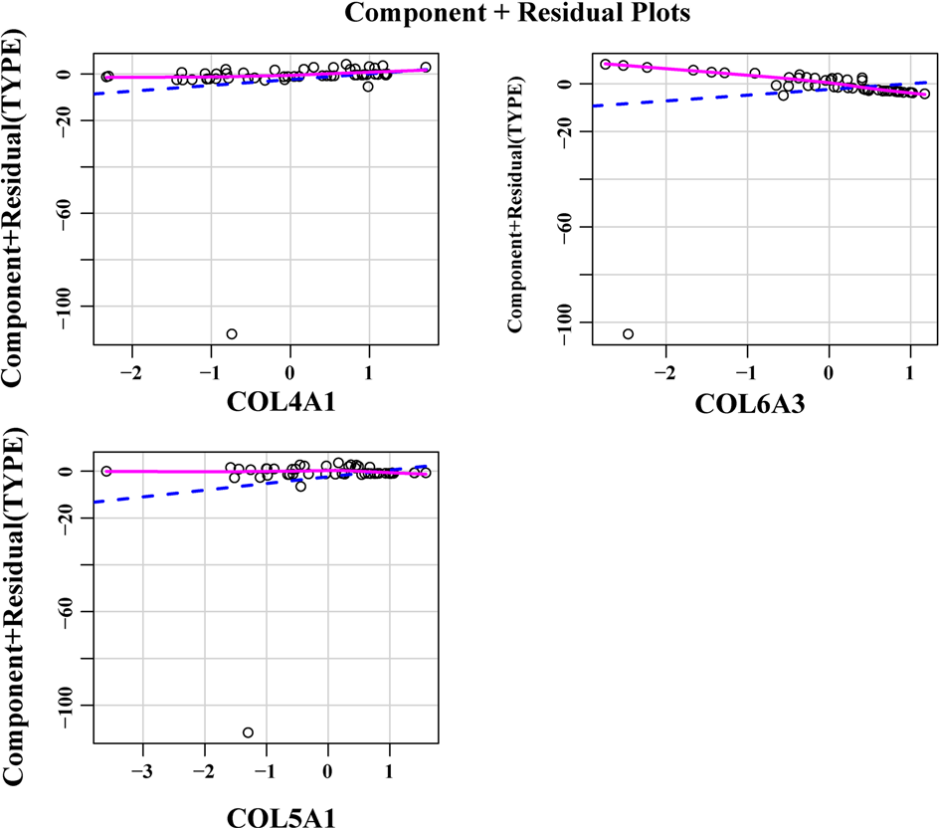
The component plus residual plot of the three genes included in the model A relatively obvious linear relationship between the horizontal axis and the vertical axis in the graph could be observed, which indicated that the independent variables were suitable for being included in the model.

**Figure 10:**
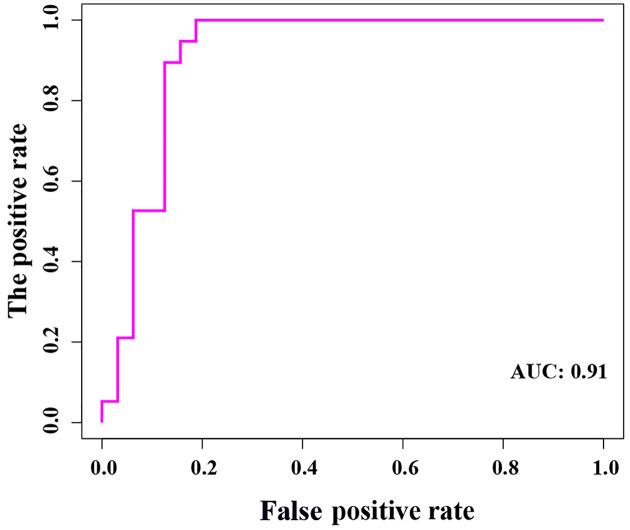
The ROC analysis results of the model

Using the three most important genes (*COL4A1, COL5A1*, and *COL6A3*), we constructed a logistic regression prediction model (Y = 0.2987 × COL5A1 + 0.9790 × COL4A1 − 2.6080 × COL6A3 − 0.6142), using the glm function in the R language. When Y ≥ 0.5, the model predicts that BM will occur in BC patients. Furthermore, we found that there was no extreme point affecting the accuracy of the model (Figure 8). All points in the model had good linear relationships (Figure 9). Further, the area under the curve (AUC = 0.91) indicated that the logistic regression prediction model—which was based on the three genes—could predict whether BM would occur in BC patients (Figure 10). Our results suggested that COL4A1, COL5A1, and COL6A3 were potentially crucial genes for the development of BCBM.

## Discussion

The development of BCBM is a multistep process that is still poorly understood. Despite advances in targeted treatment, patients with BCBM continue to exhibit an undesirable prognosis and impaired quality of life. Patients with a high risk of developing BCBM could potentially benefit from early screening strategies, as earlier diagnoses would lead to more effective BCBM treatments [30].

Thus, in the present study, we compared BC and BCBM samples in the GSE125989 and GSE100534 datasets, and first identified 146 DEGs in total. Then, based on PPI network construction, ten DEGs were selected as the most crucial genes, including *LAMA4, COL1A1, COL5A2, COL3A1, COL4A1, COL5A1, COL5A3, COL6A3, COL6A2*, and *COL6A1*. Further PCA results indicated that these genes largely contributed to the differences between BC and BCBM samples, suggesting that the ten genes were potentially important genes related to BCBM. Previous studies have demonstrated that BC could regulate the surrounding stroma or tumor microenvironments, and that various components in the BC microenvironment, including suppressive immune cells, soluble factors, and alterations in the ECM, act together to effectively impede antitumor immunity, and promote BC progression and metastasis [31–34]. In this complicated process, some key genes indeed play vital roles. Many, including *BRCA1* [23], *TP53* [35], and *Sdc1* [36] have been reported widely on. Moreover, the distinct expression patterns between BC and BCBM might be associated with the microenvironment surrounding the primary tumor, as well as its final metastatic sites [37]. Better understandings of the functional role of DEGs is of importance to identify metastasis-associated therapeutic biomarkers [38]. Thus, we performed GO and KEGG enrichment analyses on 146 DEGs. These DEGs were mainly enriched in GO terms including ECM structural constituent, ECM organization, collagen catabolic process, and extracellular region, which might be responsible for brain metastases. As previous studies have reported, the ECM which affects cancer cell characteristics is increasingly recognized as an important regulator in BC [39]. ECM in BC development features numerous changes in composition and organization when compared with the mammary gland under homeostasis [40]. Prior evidence supports our results to an extent. Additionally, most of the overlapping DEGs were enriched in KEGG pathways associated with cancer metastases, the including PI3K-Akt signaling pathway, the focal adhesion pathway, and ECM–receptor interaction pathways. Notably, it has been reported that mutations and activation of the PI3K signaling pathway in BC cells are linked to brain metastases [41]. Additionally, levels of PI3K pathway activity were significantly increased in brain metastases when compared with extracranial metastases [42]. PI3K/AKT/mTOR signaling has also been reported as one of the most important intracellular pathways, and can be considered a master regulator for cancer. However, the functional details of the overlapped DEGs still need to be further clarified.

Previous studies have demonstrated that cancer progression is highly associated with an increase in ECM deposition and stiffening [43–45]. As the major component of the ECM, collagen could increase the ability of cancer cells to reduce apoptosis, and to modulate angiogenesis which gradually promotes cancer progression [46,47]. Moreover, Gilkes et al. reported that intertumoral hypoxia could also lead to increased collagen deposition during breast cancer progression [48]. In our study, survival analyses also showed that increased expression of Collagen genes—including *COL1A1, COL14A1, COL5A1, COL6A1*, and *COL6A2*—was associated with the poor overall survival rates of BC patients. Our results support the idea that the collagen could contribute to tumor progression and result in poor prognoses. Recent studies have suggested that BCBM occurs most frequently in HER2-positive BC, with an incidence of 20–50% [49,50]. As an oncogene, HER2 may drive brain trophism, as it induces a mesenchymal state in BC cells and increases the invasiveness and metastatic potential [51]. The brain microenvironment involves several HER family ligands, including neuregulins, which result in the dimerization and activation of these receptors in metastatic brain cells [52,53]. Recent studies have demonstrated that the risk of cancer cells spreading to the brain is usually highest for women with more aggressive subtypes of BC, such as HER2 positive BC [54,55]. Thus, in this study, we analyzed the correlation between the top ten hub gene with HER2 positive expression. Our results demonstrated that *COL4A1, COL5A1*, and *COL6A3* were significantly correlated with HER2 positive expression.

Further, the TF–miRNA coregulatory network indicated that the gene expression patterns of *COL4A1, COL5A1*, and *COL6A3* were significantly correlated with each other, and they were also under the regulation of the miRNA-29 family. We speculated that those findings might provide more evidence to illustrate the underlying mechanisms of BCBM progression. Additionally, the logistic regression prediction model based on the three genes could predict whether BM could occur in BC patients, further suggesting that *COL4A1, COL5A1*, and *COL6A3* were potentially crucial genes for BCBM. Our results were consistent with some findings from previous studies. A recent study suggested that *COL4A1* was a prognostic biomarker for BC patients who had received neoadjuvant chemotherapy [56]. *COL5A1* was reported to be involved in the regulation of circRNA ACAP2 (circACAP2) in BC [57]. As for the miR-29 family, it has been reported to regulate tumor-related changes, including metastasis [58]. They might also contribute to the involvement of the multiple fibrosis process in many organs’ loss of ECM regulation, which might also be associated with the promotion of cancer cell migration and metastasis [59]. Thus, we speculated that the miR-29 family participated in the process of metastasis by modulating the cancer microenvironment. Nevertheless, exactly how the *COL4A1, COL5A1, COL6A3*, and miR-29 family are involved in the underlying mechanisms of BCBM remains to be further investigated. Moreover, the treatment value of modifying collagen conditions in BCBM is also worth exploring. Cancer resistance has substantially hindered the ability to control cancer. Therefore, both cancer cells and tumor microenvironment must be treated, and collagen is a potential target [46]. The result obtained from Awasthi’s group showed that anti-matrix metallopeptidase 9 (MMP-9) antibody therapy could decrease the COLI expression and the metastatic burden in pancreatic ductal adenocarcinoma mouse models [60]. Employing therapeutic agents directly or indirectly targeting collagen might be another promising therapeutic strategy in the treatment of cancers including BC.

There are also some limitations to our study. First, the present study was based on bioinformatics analysis of published datasets, and does not have experimental verification of the observations. Second, as we combined only two GEO datasets, the total sample size is relatively small which may result in some biasing of the results. Finally, we only analyzed the correlation between top ten hub genes with HER2 positive expression. It would also be important to investigate the correlations with other BCBM oncogenes, including estrogen receptors (ERs). Future clinical studies with larger cohorts are needed to validate our results.

## Conclusions

Here, by comparing the BC and BCBM specimens across two datasets using a series of bioinformatics analyses, we identified 146 DEGs that were potentially related to the BCBM. The DEGs were significantly enriched for ECM organization, collagen catabolic process, and cellular adhesion pathways. Among these, *COL4A1, COL5A1*, and *COL6A3* were potentially crucial genes that were associated with BCBM in BC patients. Our findings should provide more reference information for diagnostic and prognostic investigation of BCBM in the future.

## Data Availability

The datasets are available in the National Center of Biotechnology Information (NCBI) GEO (https://www.ncbi.nlm.nih.gov/geo/). Two datasets, including GSE125989 and GSE100534, were used in the present study.

## Abbreviations

BC: breast cancer
BCBM: breast cancer brain metastasis
BM: brain metastasis
BP: biological process
CC: cellular component
circACAP2: circRNA ACAP2
CNS: central nervous system
COL14A1: collagen type XIV α 1 chain
COL1A1: collagen type I α 1 chain
COL3A1: collagen type III α 1 chain
COL5A2: collagen type V α 2 chain
COL5A3: collagen type V α 3 chain
COL6A1: collagen type VI α 1 chain
COL6A2: collagen type VI α 2 chain
COL6A3: collagen type VI α 3 chain
DAVID: The Database for Annotation, Visualization and Integrated Discovery
DEG: differentially expressed gene
ECM: extracellular matrix
GEO: Gene Expression Omnibus
GEPIA: Gene Expression Profiling Interactive Analysis
GO: Gene Ontology
KEGG: Kyoto Encyclopedia of Genes and Genomes
LAMA4: laminin subunit α 4
MF: molecular function
MMP-9: Matrix metallopeptidase 9
PCA: principal component analysis
ROC: receiver operating characteristic
STRING: Search Tool for the Retrieval of Interacting Genes
TCGA: The Cancer Genome Atlas
TF: transcription factor
